# Effect of the Axial Profile of a Ceramic Grinding Wheel on Selected Roughness Parameters of Shaped Surfaces Obtained in the Grinding Process with a Dual-Tool Grinding Head

**DOI:** 10.3390/ma17102434

**Published:** 2024-05-18

**Authors:** Piotr Jaskólski, Marzena Sutowska, Wojciech Zawadka, Winfried Malorny, Krzysztof Rokosz, Krzysztof Nadolny

**Affiliations:** 1 Department of Production Engineering, Faculty of Mechanical and Energy Engineering, Koszalin University of Technology, Racławicka 15-17, 75-620 Koszalin, Poland; piotr.jaskolski@tu.koszalin.pl (P.J.); marzena.sutowska@tu.koszalin.pl (M.S.); wojciech.zawadka@tu.koszalin.pl (W.Z.); krzysztof.nadolny@tu.koszalin.pl (K.N.); 2 Faculty of Civil Engineering, Hochschule Wismar, Philipp-Müller-Straße 14, 23966 Wismar, Germany; winfried.malorny@hs-wismar.de; 3 Faculty of Electronics and Computer Science, Koszalin University of Technology, Śniadeckich 2, 75-453 Koszalin, Poland

**Keywords:** integrated machining, grinding operation, workpiece surface quality, dual-tool grinding head

## Abstract

The use of CNC equipment that integrates several machining operations eliminates downtime due to changes in setup and clamping of workpieces in more than one machining device. A review of CNC equipment and tools known from the literature and from manufacturers’ offerings indicates that new technical solutions are being developed to integrate two or more technological operations. However, these examples have numerous limitations and are mostly not suitable for machining surfaces with complex shapes. An example of such solutions is the use of a dual-tool grinding head, which integrates the process of rough grinding with a ceramic grinding wheel and finish grinding with a flexible grinding wheel. Unfortunately, it has the disadvantage of being limited by the angular shape of the ceramic grinding wheel, making it unable to adapt to the complex geometries of the shaped surfaces being ground. The need to overcome this limitation became the motivation for the research work described in this article. By means of experimental research, it was verified what effect the radial outline on the periphery of a ceramic grinding wheel realized by rough grinding would have on the surface roughness parameters obtained in the process of grinding shaped surfaces. For this purpose, grinding processes using a ceramic wheel with a conical and radial outline were compared. The result of the study was a summary of the surface roughness parameters *Sa*, *St*, *Sq*, *Spk*, *Str*, and *Sds* obtained after two-stage machining (rough and finish grinding). The obtained analysis results showed that changing the axial outline of the ceramic grinding wheel makes it possible to significantly expand the range of applications of the dual-tool head without negatively affecting the quality of the machined surface. Thus, such an improvement will make it possible to increase the applicability of the head by grinding shaped surfaces with a radial profile of curvature.

## 1. Introduction

Grinding processes ensure the achievement of high dimensional and shape accuracy and low surface roughness in workpieces. Depending on the application, they can be carried out in one or several operations, with the most common being rough grinding followed by finishing grinding (sparking-out) [1]. One example of abrasive machining carried out in this way is the grinding processes used in the manufacture of dies [2] or crankshafts [3]. Both types of grinding operations require tools of different designs (abrasive grains [4,5,6], bonds [7], structures [8], hardness [9], susceptibility [10], and elasticity [11]) and different technological parameters.

To achieve variable rough and finish grinding conditions, it is necessary to change the tool and parameter settings, which increases preparation and finishing times, analogous to machining carried out, for example, on a CNC (computerized numerical control) machining center [12] or an industrial robot [13]. These times are significantly reduced using tool magazines with automatic tool changers [14,15]. However, such solutions are costly and find justification in the case of a large series of manufactured products.

To shorten the manufacturing process, several improvements are used, including, among others, the use of hybrid machining [16,17] and integrated machining (also known as sequential or complete machining) [18,19]. To perform machining operations in this way, it is necessary to use special equipment that provides the possibility of integrating operations without changing the workpiece fixture. One such device is the DMG MORI Lasertec [20], which combines milling and laser surfacing [21,22]. Advanced tooling systems are also known to allow, for example, milling and grinding with the same head mounted in the turret of a CNC lathe with a driven *Y* axis [23].

New hybrid and integrated cavity machining methods are increasingly being studied in the literature. They are aimed at combining individual machining techniques into sequential machining, minimizing the disadvantages of the process, including reducing the energy intensity of the process, reducing machining time, and achieving better accuracy in determining the base of the workpiece. In recent years, several review articles have appeared that examine these issues more extensively [1,24]. The authors have focused on presenting the latest developments in the field of machining processes in terms of surface engineering of abrasive tools, abrasive materials, and hybridization of machining processes, especially those performed on the same machine tools. They describe solutions such as laser-assisted grinding [25], in which laser radiation heats the grinding material, plasticizing it so it can be removed more quickly by the grinding wheel, thereby improving grinding efficiency and reducing the forces acting in the grinding zone. Another example described is chemo-mechanical grinding [26], in which a chemical reaction is induced with the workpiece and integrated with mechanical material removal using an abrasive tool. Such a solution makes it possible to reduce the forces acting in the grinding zone and increase the rate of material removal while maintaining the high quality of the machined surface. The work also describes a hybrid electro-chemical process [27], in which the abrasive tool is dressed using electrochemistry instead of using the conventional method with a diamond dresser. The electrochemical process exposes new, sharp abrasive grains by dissolving grinding products from the grinding wheel during the grinding process, making such a solution less time-consuming and significantly reducing wear on the abrasive tool while keeping the workpiece roughness parameters constant.

Strategies for integrated abrasive machining using industrial robots to grind complicated free-form surfaces are also being developed [28,29]. The motivation behind this research is to reduce the high cost of carrying out the grinding process, which is usually realized with expensive precision CNC machine tools. Researchers focus on aspects of machining strategies in relation to the geometric accuracy of the workpiece. They emphasize the advantages of using industrial robots, such as manufacturing flexibility and the possibility of integrating manufacturing processes, but the implementation of the grinding process using industrial robots has a narrow range of applications at this point, as it does not provide high accuracy and repeatability of the process compared to machining on multi-axis CNC machine tools.

Composite tools with zone-differentiated structures are also being developed. These include multi-edge ball milling and grinding composite tools [30], but they are not designed for finishing and are only intended to reduce the formation of burrs in the workpiece material. Hybrid tools combining stiff and elastic grinding in a single tool pass are also known, but without the ability to control the degree of extension of the flexible grinding wheel [31], as well as abrasive tools with extendable segments for finishing grinding [32]. The solutions mentioned above can be used in the grinding of flat surfaces and do not allow the machining of surfaces with complex shape outlines.

A dual-tool grinding head has been developed at the Department of Mechanical Engineering at Koszalin University of Technology specifically for machining surfaces with complex contours in the face grinding operation of workpieces without changing their fixture [33]. The distinctive feature of the innovative head is its design, which provides a combination of rough machining with a bonded ceramic tool and finish grinding using an extendable module with a flexible coated abrasive tool. As a result, a complete machining effect (roughing and finishing) is achieved in a single workpiece fixture without the need for time-consuming tool changes.

This paper presents a continuation of research on a dual-tool grinding head, the purpose of which was to improve it by changing the shape of the axial outline of a ceramic grinding wheel in a way that would allow the variability of the shape of the machined surfaces to be extended. The aim of the research was to determine to what extent changing the axial outline of the grinding wheel would affect the roughness and morphology of the machined surface.

## 2. Dual-Tool Grinding Head

### 2.1. Design of the Head

**Figure 1:**
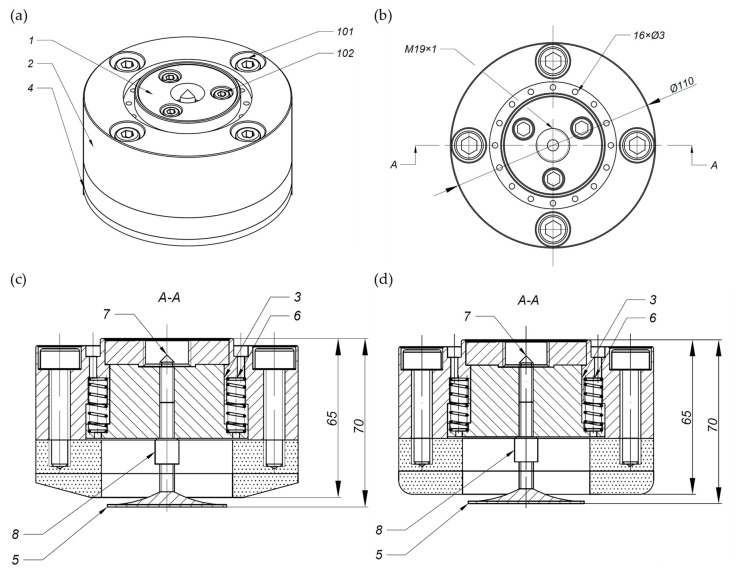
Technical drawing of a prototype of a dual-tool grinding head (1—ER14 adapter, 2—outer body, 3—inner body, 4—Ø110 grinding wheel, 5—Ø50 3M Trizact^®^ grinding wheel, 6—0.8 × 7.7 × 25 pressure spring, 7—centering shaft, 8—flexible grinding wheel spacer, 101—M8 bolt, 102—M5 bolt): (**a**) isometric view; (**b**) top view; (**c**) AA cross-section of a grinding wheel with a conical outline; (**d**) AA cross-section of a grinding wheel with a radial outline [34].

The tool that was used for the study was a dual-tool grinding head consisting of a ceramic wheel for rough grinding and a flexible wheel for finish grinding. The advantage of this solution is that it allows rough and finish grinding on a single machine tool and in a single workpiece fixture. The outline of the ceramic grinding wheel can be conical or radial, depending on the need to adapt it to the machining of shaped surfaces with a certain curvature. This paper compares grinding processes using a 7 mm thick ceramic grinding wheel shaped to a conical outline at a 15-degree angle from a diameter of 110 mm to 55 mm, more extensively presented in the article [34], and a 7 mm thick ceramic wheel shaped to a radial outline with a radius of 6 mm and a diameter of 110 mm with a bore of 55 mm (Figure 1).

**Figure 2:**
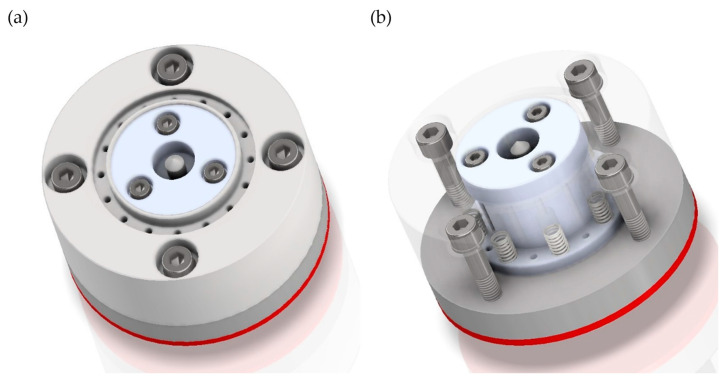
The 3D digital model of a dual-tool grinding head: (**a**) external body view; (**b**) internal body view.

During the design of the head, several changes were made to the patent-protected solution [34]. Among other things, the method of mounting the ceramic grinding wheel was changed so that it is not fused to the body but is screwed onto threaded inserts, which allows faster replacement of worn grinding wheels. In addition, the number of pressure springs was increased, and the method of extending the head was changed (instead of a thrust bearing located directly on the head, rollers mounted on pneumatic cylinders have been used, thus significantly reducing the weight of the tool). The prototype head consists of three basic components: an outer body, an inner body, and a mounting adapter. All components are connected by springs, bolts, and nuts to mount a ceramic grinding wheel on the outer body and a flexible wheel on the inner body (Figure 2).

**Figure 3:**
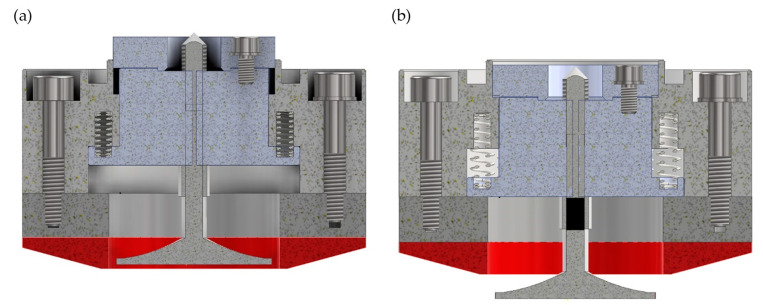
The 3D digital model of a dual-tool grinding head with a conical outline grinding wheel: (**a**) in the rough grinding setting; (**b**) in the finishing grinding setting.

**Figure 4:**
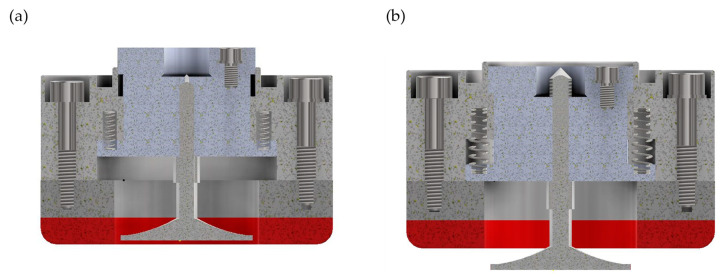
The 3D digital model of a dual-tool grinding head with a radial outline wheel: (**a**) in the rough grinding setting; (**b**) in the finishing grinding setting.

Figure 3 and Figure 4 show a cross-sectional view of the digital 3D model of the grinding head in two positions for two ways of shaping the axial outline (conical—Figure 3 and radial—Figure 4). The figures show the head in the position for rough grinding with a ceramic grinding wheel (conical outline—Figure 3a, radial outline—Figure 4a) and in the position for finishing grinding with an extended flexible grinding wheel (conical outline—Figure 3b, radial outline—Figure 4b).

During the grinding process, as the geometry of the ceramic grinding wheel and the geometry of the machined surface change, the cross-section of the grinding layer can change significantly. Therefore, it is important to properly select the shape of the grinding wheel for the machined surface. A grinding wheel with a conical outline adapts to the shape of workpieces with an angular surface outline, but if one were to apply these wheel shapes to surfaces with a radial outline, there would be excess material left over after the grinding process, which would not be removed due to the geometric limitation. Therefore, by using a ceramic grinding wheel shaped to a radial outline, we have greater control over the cut cross-section of shaped surfaces. For a grinding wheel with a radial outline, we get a greater fit to the machined surface, so this parameter allows greater control over the cross-section of grinding layers and affects process stabilization, tool life, and the quality of the variable geometries of the machined surfaces. Thus, it is possible to grind not only oblique surfaces but also curved surfaces with varying curvature. A more extensive analysis of the cross-sections of grinding layers is presented in the chapter on research methodology.

### 2.2. Characteristics of Grinding Wheels

**Figure 5:**
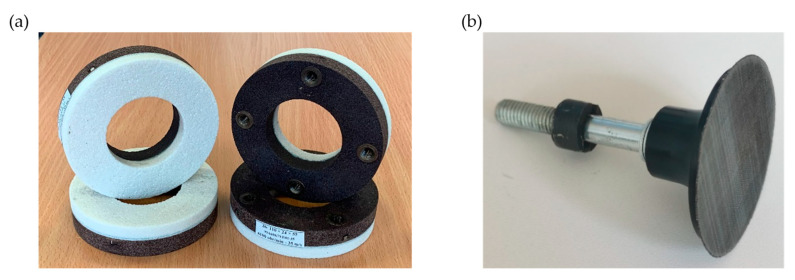
Work components of a dual-tool grinding head: (**a**) a ceramic grinding wheel from Andre Abrasive Articles (Kolo, Poland), type 3611-110 × 24 × 55-99A60K9V with four M8 threaded inserts; (**b**) a flexible grinding wheel with Trizact^®^ grains from 3M (Wroclaw, Poland) company.

For both ways of shaping the ceramic grinding wheel in the rough grinding process, a grinding wheel made by Andre Abrasive Articles (Kolo, Poland) was used (Figure 5a). Flexible coated abrasive tool Trizact^®^ grains from 3M (Wroclaw, Poland) company was used to realize finish grinding [35] (Figure 5b). Table 1 provides key technical information on the abrasive tools applied.

**Figure 6:**
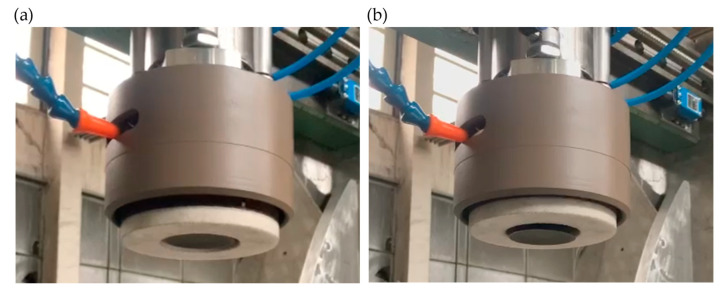
Dual-tool grinding head: (**a**) in the rough grinding mode; (**b**) in the finishing grinding mode.

The head is also shown in a working arrangement with the actuators extended, i.e., the position for realizing the rough grinding function (Figure 6a), and with the ceramic grinding wheel withdrawn, i.e., the position for realizing finishing grinding using a flexible coating tool (Figure 6b).

## 3. Research Methodology

The purpose of the study was to determine to what extent a change in the axial outline of a ceramic grinding wheel would affect the roughness and morphology of the machined surface. The study examined how the grinding process would be affected by shaping the ceramic grinding wheel into a conical or radial outline. To achieve the goal, it was necessary to:

- Prepare the test stand.
- Shape the ceramic grinding wheels.
- Design the workpieces, including material selection.
- Carry out milling, rough grinding, and finishing grinding processes.
- Perform microtopography analysis of the machined surface using a Hommel-Tester T8000 (Hommelwerke GmbH, Villingen-Schwenningen, Germany) contact profilometer and a Taylor-Hobson (Leicester, UK) Talysurf CLI 2000 multi-head measurement system.
- Conduct analysis of the machined surface morphology and active surface of abrasive tools using a Keyence International NV/SA (Mechelen, Belgium) VHX7000 digital microscope and a Quanta 250 FEI Company (Eindhoven, The Netherlands) scanning electron microscope.

**Figure 7:**
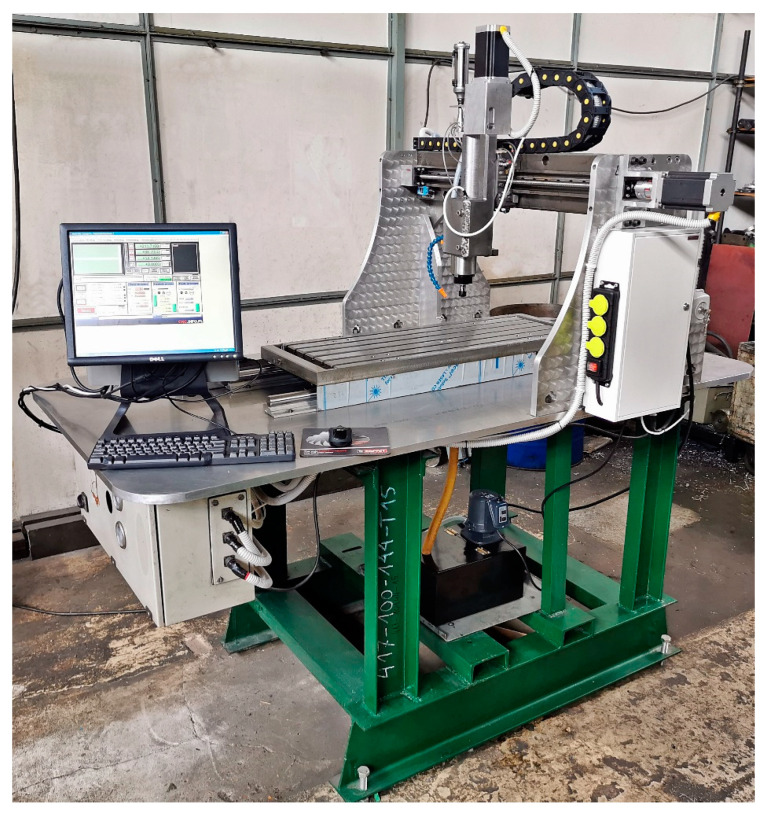
Prototype device integrating spatial scanning, milling, and grinding of shaped surfaces.

Studies of the grinding process using a dual-tool grinding head were carried out on a prototype device integrating spatial scanning, milling, and smoothing of contoured surfaces (Figure 7). The CNC device was designed and manufactured by employees of the Mechanical Engineering Laboratory Group of the Mechanical Engineering Department of the Koszalin University of Technology. The described device enables spatial scanning, 3-axis milling, and grinding operations, and a more extensive description of it is presented in the article [36].

**Figure 8:**
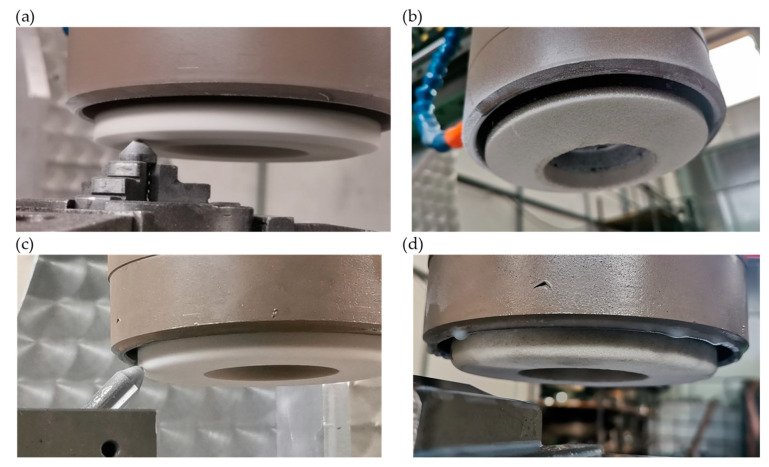
Dressing of the ceramic grinding wheel: (**a**) the process flow of shaping a conical ceramic grinding wheel with a diamond dresser; (**b**) shaped ceramic grinding wheel with a conical outline; (**c**) the process flow of shaping a radial ceramic grinding wheel with a diamond dresser; (**d**) shaped ceramic grinding wheel with a radial outline.

The conducted research began with shaping the axial outlines (conical and radial) of ceramic grinding wheels in a dressing procedure. For this purpose, using Autodesk Inventor Professional 2023 with CAM Ultimate 2023 software (Mill Valley, CA, USA), a machine tool control G-code was developed to shape the grinding wheel with a diamond dresser at an angle of 15° for the wheel with a conical outline and with a radius of 6 mm for the wheel with a radial outline. The dressing parameters of the grinding wheel were constant for shaping both outlines; that is, the grinding wheel speed was 15,000 rpm, the feed speed was 1000 mm/min, and the dressing layer of the grinding wheel was 0.02 mm. The result of the work is shown in Figure 8.

**Figure 9:**
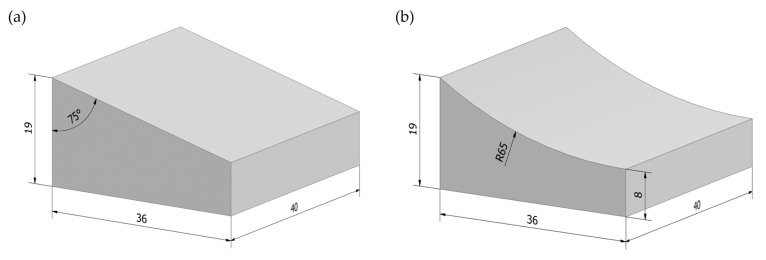
The 3D digital model of machined workpieces: (**a**) angular workpiece machined with a conical outline grinding wheel; (**b**) radial workpiece machined with a radial outline grinding wheel (dimensions given in mm).

To conduct the tests, workpieces were designed, which are shown in Figure 9. Two workpiece geometries were designed to verify that a grinding wheel with a radial outline would enable the grinding process to be carried out on workpieces with a radial surface outline with the same accuracy as a grinding wheel with a conical outline would enable the grinding of workpieces with an angular surface outline. Figure 9a shows the geometry of the workpiece obtained in the grinding process using a grinding wheel with a conical wheel outline. In turn, Figure 9b shows the geometry of the workpiece obtained in the grinding process using a grinding wheel with a radial wheel outline.

Tool steel 1.2510, 100MnCrW4 (PN NMWV), was used for both workpiece geometries. It is a steel with good cutting properties, characterized by dimensional stability during heat treatment as well as good hardenability. It was chosen because of its wide area of application, including in the manufacture of molds, dies, stamping dies, jaws, machine knives, or measuring instruments. The characteristics of tool steel 1.2510 are shown in Table 2.

The geometries of the shaped workpieces were obtained by three machining processes: milling, rough grinding, and finish grinding.

In the first stage of machining both designed workpieces, a milling process was carried out using a ball mill with a diameter of 20 mm at a rotational speed (*n*) of 3900 rpm, a feed rate (*v_f_*) of 600 mm/min, a cutting width (*a_e_*) of 1 mm, and a cutting depth (*a_p_*) in the range of 1–5 mm.

In the second stage of machining the two components, a rough grinding process was carried out using ceramic grinding wheels with a conical and radial outline using the following parameters: rotational speed (*n*) of 8100 rpm, feed rate (*v_f_*) of 1000 mm/min, and grinding depth (*a_p_*) of 0.04 mm. The variable, however, was the grinding width (*a_e_*), which was 2 mm for the conical outline and 0.5 mm for the radial outline. Changing this parameter was necessary due to the constraints imposed by the geometry of the grinding wheel’s axial outline.

**Figure 10:**
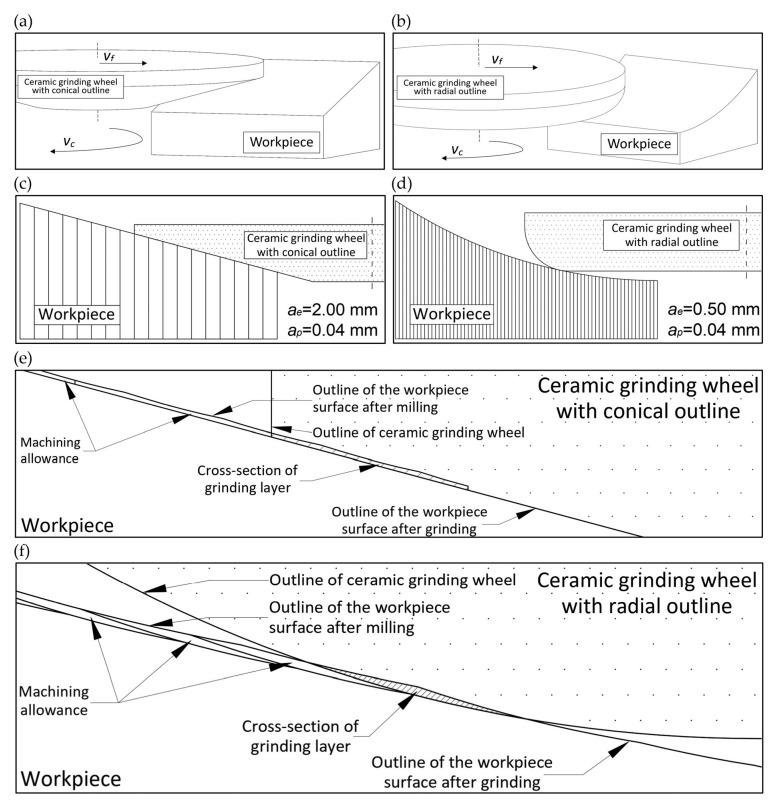
Grinding operation concept: (**a**) indication of the direction of tool rotation and the direction of tool feed relative to the workpiece material for grinding with a conical grinding wheel; (**b**) indication of the direction of tool rotation and the direction of tool feed relative to the workpiece material for grinding with a radial grinding wheel; (**c**) indication of fitting the tool shape to the workpiece material for grinding with a conical grinding wheel; (**d**) indication of fitting the tool shape to the workpiece material for grinding with a radial grinding wheel; (**e**) enlarging the grinding zone of the material using a conical grinding wheel specifying the cut material; (**f**) enlarging the grinding zone of the material using a radial grinding wheel specifying the cut material.

Figure 10 shows the adopted machining concept for a rough grinding process using a ceramic grinding wheel.

**Figure 11:**
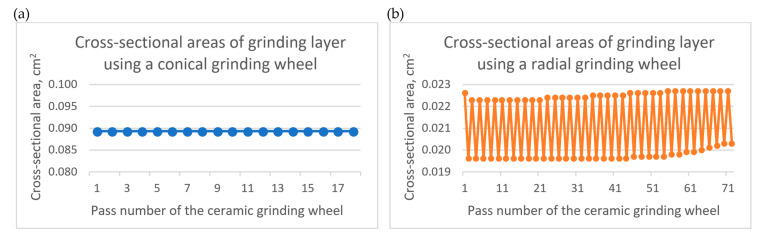
Cross-sectional areas of the grinding layer of the workpiece material in individual passes of the rough grinding operation using a ceramic grinding wheel with (**a**) a conical outline and (**b**) a radial outline.

Figure 10a,b shows the direction of rotation of the head and in which direction the head moves relative to the workpiece. Figure 10c,d shows how the ceramic grinding wheel adapts to the workpieces depending on the shape of the wheel and the workpiece. In addition, the vertical lines indicate the grinding width of the workpieces (parameter *a_e_*). Figure 10e,f shows the concept of rough grinding under magnification. The outlines of the machined surfaces obtained by milling and grinding are marked, and the cross-sectional area of the grinding layer in the individual passes of the ceramic grinding wheel is calculated on this basis. For the grinding process using a ceramic grinding wheel with a conical outline, it was calculated that in each pass (out of 18 passes of the grinding wheel with the parameter *a_e_* = 2 mm), the cross-sectional area of the grinding layer during rough grinding was constant at 0.0893 cm^2^. On the other hand, for the grinding process using a wheel with a radial outline, it was calculated that in each pass (from 72 passes of the grinding wheel with the parameter *a_e_* = 0.5 mm), the cross-sectional area of the grinding layer during rough grinding varied and ranged from 0.0195 to 0.0227 cm^2^. The detailed values of the cross-sectional areas of the grinding layer in individual passes of the ceramic grinding wheel with a conical and radial outline are shown in Figure 11.

In the final, third stage of workpiece machining, a finishing grinding process was carried out with a flexible grinding wheel using a rotational speed (*n*) of 15,000 rpm, a feed rate (*v_f_*) of 1000 mm/min, and a grinding width (*a_e_*) of 2 mm. In this case, the variable was the grinding depth (*a_p_*), which was 0.1 mm for the workpiece obtained by grinding with a conical grinding wheel and 0.5 mm for the workpiece obtained by grinding with a radial grinding wheel. The depth of grinding in the workpiece with a radial outline had to be increased due to a change in the geometry of the machined surface, to which the flexible grinding wheel had to adjust to a greater extent than in the workpiece with a tapered outline. A summary of the various machining parameters is shown in Table 3 and Table 4.

**Figure 12:**
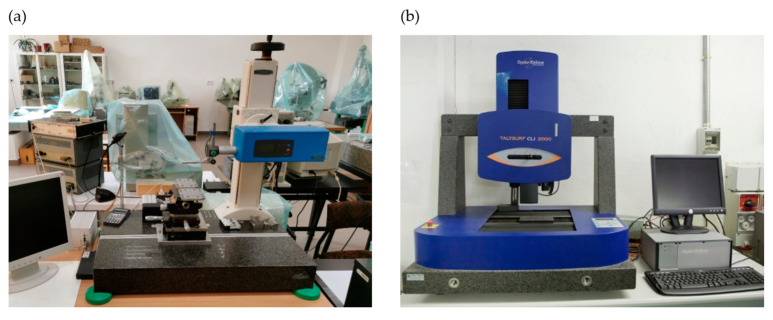
Measuring stations: (**a**) Hommel Tester T8000 contact profilometer from Hommelwerke and (**b**) Taylor-Hobson Talysurf CLI 2000 multi-head measuring system.

Microtopographic analysis of surfaces machined with a conical-shaped grinding wheel was carried out using a Hommel-Tester T8000 contact profilometer from Hommelwerke (Hommelwerke GmbH, Villingen-Schwenningen, Germany) (Figure 12a), while analysis of surfaces machined with a radial-shaped grinding wheel was carried out by optical method using a Taylor-Hobson (Leicester, UK) Talysurf CLI 2000 multi-head measuring system (Figure 12b). The change in method was necessary due to the limitation of the contact profilometer, which did not have a sufficient measurement range for the prepared workpiece with a contoured surface.

A Hommel Tester T8000 contact profilometer from Hommelwerke (Hommelwerke GmbH, Villingen-Schwenningen, Germany) was used to measure the geometric structure of surfaces machined in three machining stages using a conical grinding wheel. It is a device that allows the measurement of standard profile parameters such as roughness or waviness. When obtaining a measurement of the topography of the surface to be measured, the device precisely moves the test piece along the Y-axis of the object to be measured by means of a sliding measuring table. The device allows a maximum measurement of 120 mm at a speed of 0.05 to 0.5 mm/s. The profilometer consists of a Waveline 60 Basic drive unit (Hommelwerke GmbH, Villingen-Schwenningen, Germany), a Wavelift 400M measuring column (Hommelwerke GmbH, Villingen-Schwenningen, Germany), and a Wavesystem 780 granite table base (Hommelwerke GmbH, Villingen-Schwenningen, Germany). In addition, the device is controlled by Turbo Roughness 3.44 and Hommel Map Basic 3.0.8 software.

During the tests, a TKL 100/17 needle sensor (Hommelwerke GmbH, Villingen-Schwenningen, Germany) was used, allowing measurements within a range of ±100 µm, a nominal tip rounding radius of 2 µm, a tip angle of 90°, and a tip load of 0.5 N applied vertically to the surface.

Measurements of the geometric structure of surfaces machined with a conical grinding wheel were carried out for surfaces obtained in three stages of machining. During the measurement, a microtopography was recorded on each sample with dimensions (*x*, *y* axis) of 15 × 15 mm^2^. The lamellar section was 2.5 mm, and the measurement was carried out at a speed of 0.5 mm/s. Each measurement taken consisted of measuring 501 profiles (*y*-axis). The distance between each profile was equal to 30 μm. On a single profile, 9600 points were recorded (*x*-axis). The distance between the points of the profile was 1.56 μm. Each measurement was carried out in single-pass mode. The measurement time of one microtopography was 500 min.

On the other hand, to measure the geometric structure of surfaces machined in three stages of machining with a radial outline grinding wheel, a measuring station was used, equipped with a high-tech Talysurf-type CLI 2000 optical multiprofilometer manufactured by Taylor-Hobson (Leicester, UK). The working range of this device (*x*, *y* axis) is 200 × 200 mm^2^. With the Talysurf optical multiprofilometer type CLI 2000, it is possible to test the surfaces of samples with a height (*z*-axis) of up to 200 mm whose mass does not exceed 15 kg. The multiprofilometer is equipped with three measuring sensors: an inductive sensor, a CLA-type head, and an optical (laser) sensor type LK-031. Microtopography measurements of the geometric structure of the surface of elements were carried out by a non-contact optical method using a laser sensor type LK-031, whose manufacturer is Keyence Corp. (Osaka, Japan). Using this sensor, it is possible to record the height of surface irregularities up to 10 mm with a resolution of 0.5 µm. This sensor makes it possible to take accurate measurements at speeds of up to 30 mm/s.

Measurements of the geometric structure of surfaces machined with a radial outline grinding wheel were carried out for surfaces obtained in three stages of machining. During the measurement, microtopography was recorded on each sample with dimensions (*x*, *y* axis) of 15 × 15 mm^2^. Each measurement performed on the optical multiprofilometer consisted of measuring 501 profiles (*y*-axis). The distance between each profile was equal to 30 μm. On a single profile, 10,001 points were recorded (*x*-axis). The distance between the points of the profile was 1.5 μm. Each measurement was carried out in single-pass mode. The measurement time of one microtopography was 280 min, and the results were processed in Taly-Map Silver 4.1.2 software (Digital Surf, Besançon, France).

Based on the obtained measurement results, a graphical presentation of the microgeometry of the measured areas was prepared, and the values of surface texture parameters were determined. A Gaussian filter was used to calculate the values of the surface geometric structure roughness parameters. When analyzing the results, the most significant surface roughness parameters were determined:

- *Sa*—arithmetic mean deviation of the surface
- *St*—total height of the surface
- *Sds*—density of summits of the surface
- *Sq*—root-mean-square deviation of the surface
- *Str*—texture aspect ratio of the surface
- *Spk*—reduced peak height

The adopted collection of parameters allows multi-criteria parametric evaluation of the machined surface texture by considering parameters belonging to the group of amplitude parameters (*Sa*, *St*, *Sq*), spatial parameters (*Str*, *Sds*), and the Abbott-Firestone Curve parameter (*Spk*). The selected parameters provide both geometric and functional (bearing capacity) characteristics of the machined surfaces under evaluation.

**Figure 13:**
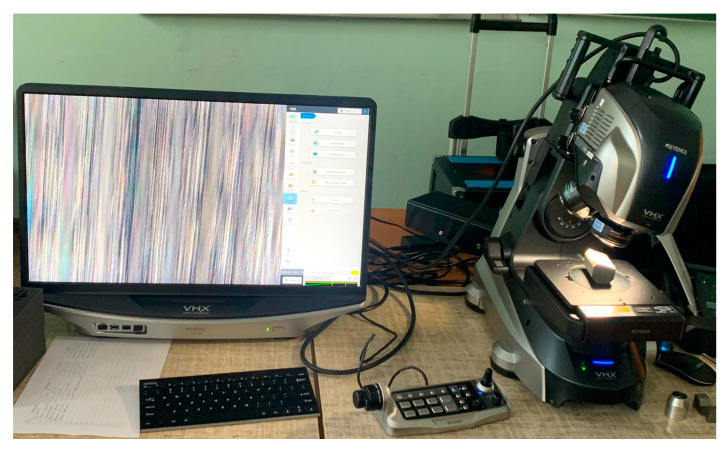
Measurement position with the digital microscope VHX7000 from Keyence International NV/SA (Mechelen, Belgium).

A VHX7000 digital microscope (Figure 13) from Keyence International NV/SA (Mechelen, Belgium) was used to analyze the surface morphology of ceramic grinding wheels, flexible grinding wheels, and the surfaces of machined objects. The device features a progressive scanning system, a 1/1.7-inch, 12.22-megapixel CMOS sensor, a maximum frame rate of 30 fps, and a maximum resolution of 12,000 × 9000. In addition, the microscope has a number of specialized functions, such as auto-focus function, working distance preview function, illumination switch function (full, partial, side, dark field, bright field, mixed illumination), automatic vibration correction function, glare removal function, and 2D and 3D image fusion. Thanks to its use, it is possible to obtain several measurements, such as distances, angles, radii, area, and analysis of grain size or impurities. During the study, microscopic views of machined surfaces at 400× magnification, views of active surfaces of ceramic grinding wheels at 80× magnification, and active surfaces of flexible grinding wheels with Trizact^®^ grains at 20× magnification were recorded.

The Quanta 250 FEI Company (Eindhoven, The Netherlands) scanning electron microscope, equipped with Low Vacuum and ESEM modes and featuring a field emission cathode, along with the Noran System Six energy dispersive X-ray spectroscopy (EDX) system employing a nitrogen-free silicon drift detector, was also used for the analysis of workpieces and grinding tool surface morphology.

## 4. Results and Discussion

**Figure 14:**
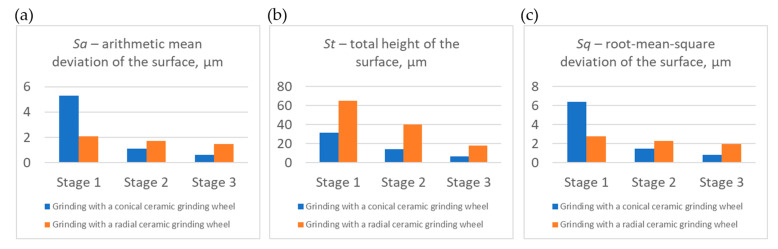
Values of selected surface roughness parameters after milling (Stage 1), after milling and rough grinding (Stage 2), and after milling, rough grinding, and finish grinding (Stage 3) using a conical outline grinding wheel and a radial outline grinding wheel: (**a**) *Sa*—arithmetic mean deviation of the surface; (**b**) *St*—total height of the surface; (**c**) *Sq*—root-mean-square deviation of the surface.

**Figure 15:**
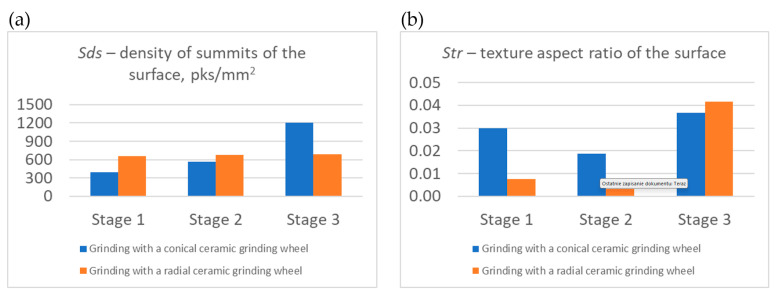
Values of selected surface roughness parameters after milling (Stage 1), after milling and rough grinding (Stage 2), and after milling, rough grinding, and finish grinding (Stage 3) using a conical outline grinding wheel and a radial outline grinding wheel: (**a**) *Sds*—density of summits of the surface; (**b**) *Str*—texture aspect ratio of the surface.

**Figure 16:**
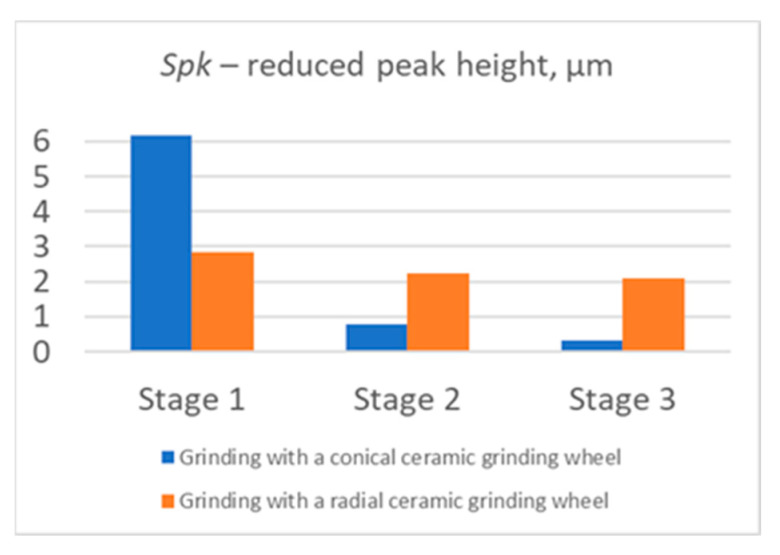
Reduced peak height *Spk* of the Abbott-Firestone curve after milling (Stage 1), after milling and rough grinding (Stage 2), and after milling, rough grinding, and finish grinding (Stage 3) using a conical and radial outline grinding wheel.

The obtained results of measurements of selected roughness parameters (*Sa*, *St*, *Sds*, *Sq*, *Str*, and *Spk*) generated in three stages of machining of the carried-out process with a conical and radial outline grinding wheel are summarized in Figure 14, Figure 15 and Figure 16. The analysis of selected roughness parameters was divided into groups of amplitude parameters (Figure 14), spatial parameters (Figure 15), and the Abbott-Firestone load curve parameter (Figure 16). The measured values of the roughness parameters are marked in blue for the process carried out with a conical outline grinding wheel and in orange for the process carried out with a radial outline grinding wheel. Table 5 shows the exact values of the obtained parameters, which are presented in charts.

The group of amplitude parameters (Figure 14), relating to the values of the average ordinates *Sa*, *St*, and *Sq*, showed a decrease in the successive values of the parameters, as evidenced by:

- A decrease in the arithmetic mean height of 5.30 μm, 1.12 μm, and 0.63 μm, respectively, for the process carried out with a conical grinding wheel, and a decrease of 2.10 μm, 1.73 μm, and 1.48 μm for the process carried out with a radial grinding wheel.
- Reduction in the total height of the surface profile of 31.8 μm, 14.4 μm, and 6.72 μm, respectively, for the process carried out using a conical outline grinding wheel, and 65.0 μm, 40.4 μm, and 18.3 μm for the process carried out using a radial outline grinding wheel.
- Decreasing the mean square deviation of the surface by 6.39 μm, 1.46 μm, and 0.81 μm, respectively, for the process carried out with a conical outline grinding wheel, and 2.78 μm, 2.29 μm, and 1.97 μm for the process carried out with a radial outline grinding wheel.

This means that the evaluated surfaces in each successive machining step in both variants had increasingly lower roughness and were thus smoothed more and more.

In the group of spatial parameters (*Sds* and *Str*), shown in Figure 15, the value of the *Sds* parameter, which informs about the density of surface vertices, increased in successive machining stages (393 pks/mm^2^, 563 pks/mm^2^, and 1203 pks/mm^2^ for the process carried out with a conical outline grinding wheel, and 654 pks/mm^2^, 678 pks/mm^2^, and 686 pks/mm^2^ for the process carried out with a radial outline grinding wheel). This means that although the number of peaks increased, they are much smaller, as indicated by the amplitude parameters—increasingly lower levels of roughness and more peaks. On the other hand, the value of the *Str* parameter, which describes the degree of directionality of the surface, decreased after the second stage (for the process carried out with a conical-oriented grinding wheel from 0.0300 to 0.0187 and for the process carried out with a radial-oriented grinding wheel from 0.008 to 0.003), and after the third stage it increased significantly (for the process carried out with a conical grinding wheel from 0.0187 to 0.0367 and for the process carried out with a radial grinding wheel from 0.003 to 0.040). This is since the first stage of the milling process produced machining traces with a directed shape (transverse–longitudinal) resulting from the trajectory of movement of the individual cutter blades and the value of the parameters, mainly the feed per tooth. In the second stage of machining, the value of the Str parameter decreased as rough machining with a ceramic grinding wheel smoothed out transverse irregularities and left only longitudinal machining traces. In the last stage, the value of the Str parameter increased relative to the first stage because finishing with the flexible grinding wheel left multidirectional machining traces resulting from tool dimension, feed, rotation, and the intersecting paths of the Trizact^®^ abrasive grain directions.

In addition, one of the parameters of the Abbott-Firestone curve, *Spk*, was selected (Figure 16). This parameter was chosen for its best imaging of the upper surface apical zone. Its values also decreased in the successive stages of machining (6.16 μm, 0.77 μm, and 0.33 μm for the process carried out with a conical grinding wheel, and 2.85 μm, 2.23 μm, and 2.09 μm for the process carried out with a radial grinding wheel), which indicates a reduction in the top height of the machined surface. In the results obtained, there were no clear differences between the use of a ceramic grinding wheel with a tapered and radial curvature outline, which means that the limitations of the shape of the grinding wheel are eliminated. As a result, it is possible to carry out machining of curvilinear surfaces with varied curvature, not only angled surfaces.

**Figure 17:**
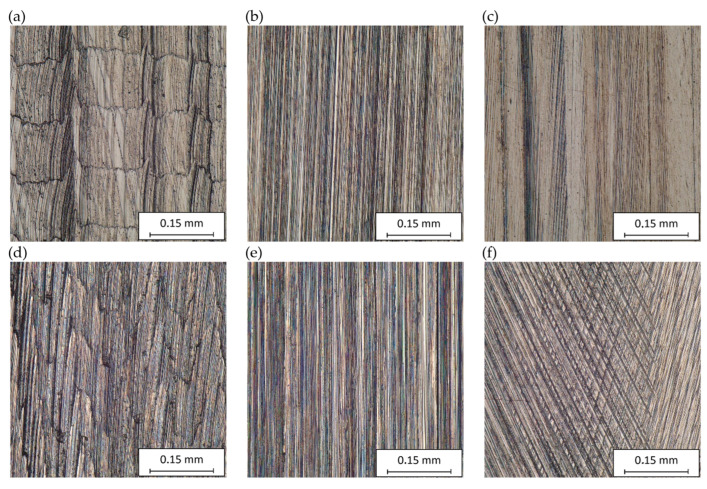
Microscopic view of the machined surface at 400× magnification: (**a**) view after contour milling of an angular surface; (**b**) view after contour milling and rough grinding of an angular surface; (**c**) view after contour milling, rough grinding, and finish grinding of an angular surface; (**d**) view after contour milling of radial contour surfaces; (**e**) view after contour milling and rough grinding of radial contour surfaces; (**f**) view after contour milling, rough grinding, and finish grinding of radial contour surfaces.

**Figure 18:**
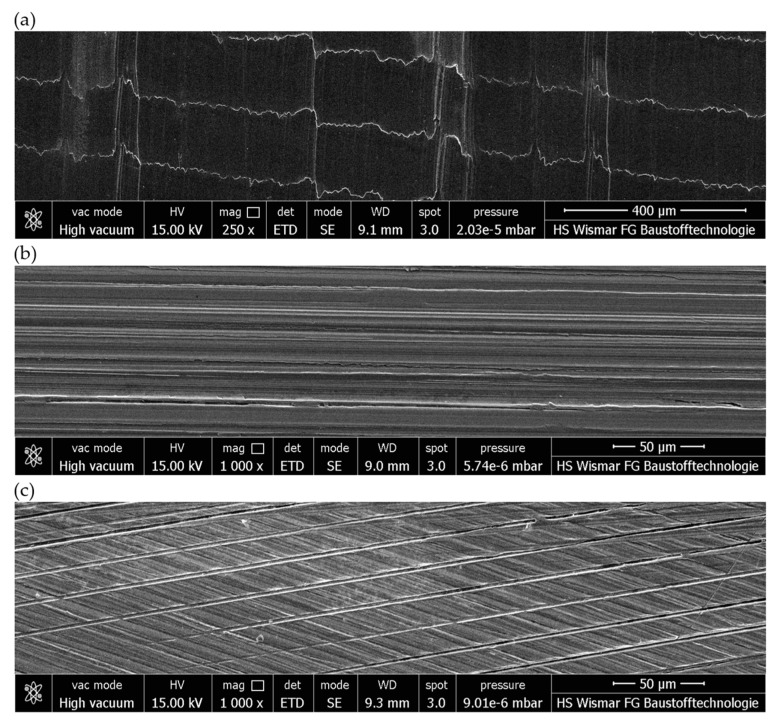
Images obtained using the scanning electron microscope: (**a**) view after contour milling of an angular surface; (**b**) view after contour milling and rough grinding of an angular surface; (**c**) view after contour milling, rough grinding, and finish grinding of an angular surface.

Figure 17 and Figure 18 show the results of the analysis of the morphology of the machined surfaces. The results of the analysis presented are aimed at evaluating the visual aspects of the machined surfaces obtained in two variants of grinding wheel shape.

Figure 17a–c and Figure 18a–c show the machined surfaces obtained in three stages of machining in the grinding process with a conical-shaped grinding wheel, and Figure 17d–f shows the machined surfaces obtained in three stages of machining in the grinding process with a radial-shaped grinding wheel. The images show differences due to the use of different tools and different machining parameters. The first stage (Figure 17a,d and Figure 18a) shows machining marks with a directed shape, resulting from the trajectory of the movement of individual cutter blades and the values of the parameters, mainly the feed per tooth. In the second stage (Figure 17b,e and Figure 18b), the ceramic grinding wheel smoothed out the transverse irregularities and left only longitudinal machining marks. On the other hand, in the last, third stage (Figure 17c,f and Figure 18c), machining with the flexible grinding wheel left multidirectional machining traces shaped by intersecting paths resulting from tool dimension, feed, rotation, and directions of movement of Trizact^®^ abrasive grains. The visible machining traces confirm the conclusions drawn from the analysis of selected roughness parameters.

**Figure 19:**
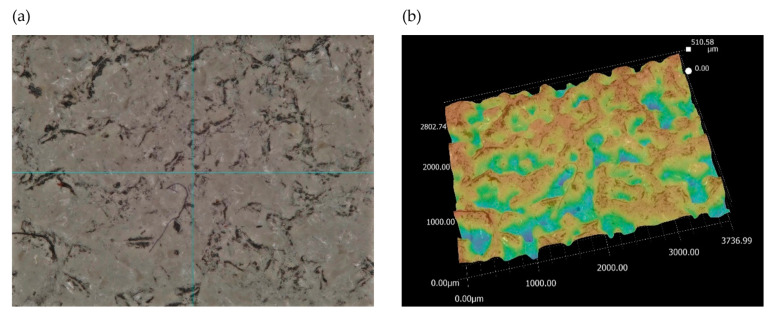
Analysis of the active surface of a conical ceramic grinding wheel at 80× magnification: (**a**) microscopic view and (**b**) topography analysis.

**Figure 20:**
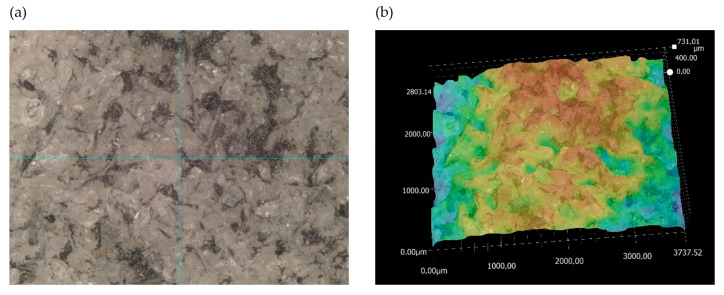
Analysis of the active surface of a ceramic grinding wheel with radial outline at 80× magnification: (**a**) microscopic view and (**b**) topography analysis.

Next, the analysis of the morphology of the active surfaces of the grinding wheels proceeded. First, ceramic grinding wheels with conical and radial outlines were analyzed (Figure 19 and Figure 20).

**Figure 21:**
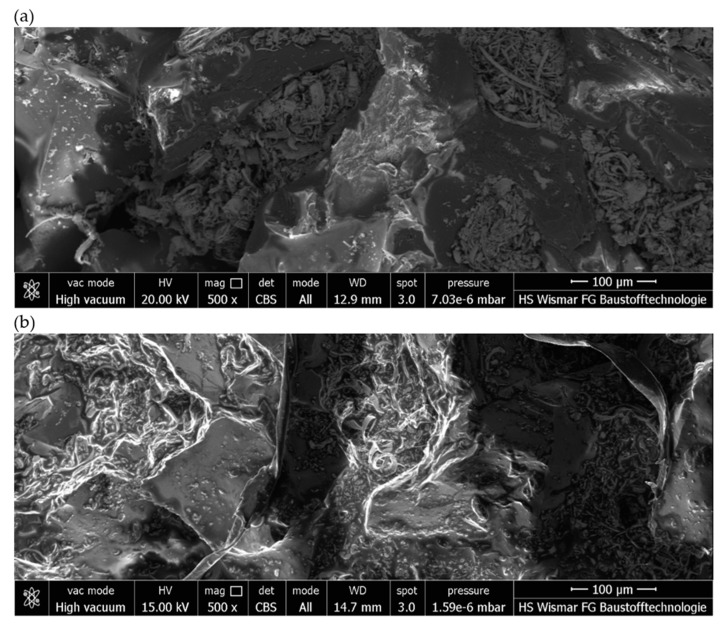
Images obtained using the scanning electron microscope of selected fragments of a conical ceramic grinding wheel at 500× magnification: (**a**) fragment 1 and (**b**) fragment 2.

The images show how the way the grinding wheels were shaped affected the grinding process. For a grinding wheel with a conical outline, the wheel machined the surface of the workpiece uniformly along a slice of the wheel’s linear cone. On the other hand, in a grinding wheel with a radial outline, it is apparent that the grinding wheel machined the surface of the workpiece unevenly, that is, only along the periphery of a portion of the radial outline. Images obtained using the scanning electron microscope (Figure 21) show the proportion of free inter-grain spaces where the products of the grinding process, such as chipped grinding wheel grains, ribbon chips, and many micrographs, have been anchored. In addition, individual microcalcifications are visible on the tops of the active abrasive grains. This is most likely due to the insufficient openness of the grinding wheel structure or insufficient coolant and lubricant in the cutting zone. The analysis of the presented images shows that it is necessary to dress the grinding wheel and reshape it to the desired shape before grinding further test samples.

**Figure 22:**
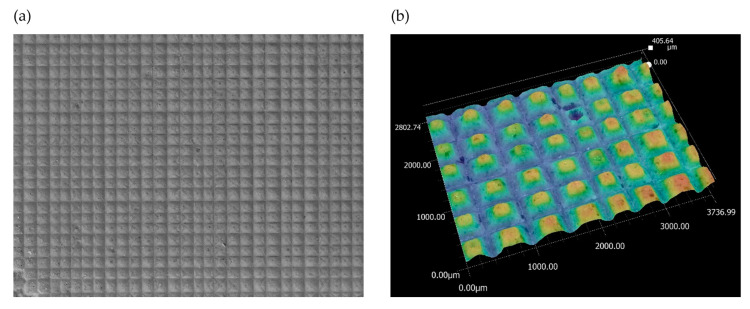
Analysis of the active surface of a flexible grinding wheel with Trizact^®^ grains after the grinding process using a ceramic grinding wheel with a conical outline at 20× magnification: (**a**) microscopic view and (**b**) topography analysis.

**Figure 23:**
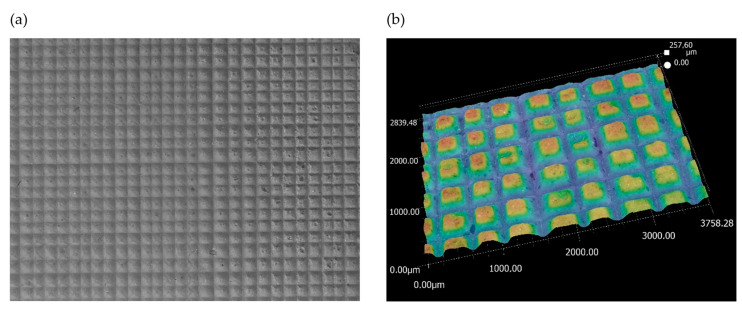
Analysis of the active surface of a flexible grinding wheel with Trizact^®^ grains after the grinding process using a ceramic grinding wheel with a radial outline at 20× magnification: (**a**) microscopic view and (**b**) topography analysis.

**Figure 24:**
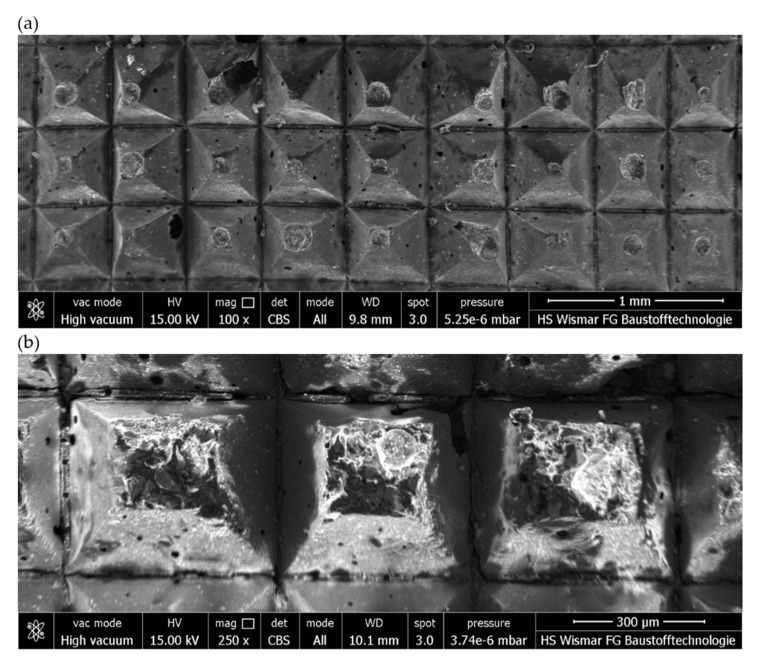
Images obtained using the scanning electron microscope of a flexible grinding wheel after the grinding process using a ceramic grinding wheel with a conical outline: (**a**) at 100× magnification and (**b**) at 250× magnification.

In the next stage of the work, the active surfaces of flexible grinding wheels were subjected to morphological analysis after the finishing grinding of surfaces machined with conical and radial grinding wheels (Figure 22 and Figure 23). The analysis of the active surfaces of flexible grinding wheels shows that the Trizact^®^-type grains are only moderately worn. The recorded images show isolated chipping of the grains, which means endurance wear, but it is relatively small, visible only on the tops of the active abrasive grains (Figure 24). This shows that the grinding wheel has not lost its cutting ability and can be used for grinding subsequent components.

The analysis showed that changing the axial outline of the ceramic grinding wheel makes it possible to significantly expand the range of applications of the dual-tool head without adversely affecting the quality of the machined surface. It was proven that the appropriate selection of the shape of the ceramic grinding wheel for the machined surface had a positive effect on the process. A similar geometric match was obtained for the grinding wheel with a radial contour to the surface with a radial contour, compared to the match of the grinding wheel with a conical contour to the machined surface with an angular contour workpiece. By using a ceramic grinding wheel shaped to a radial outline, greater control over the machined cross-section of the shaped surfaces was achieved, and therefore the change in this parameter had a positive effect on process stabilization, tool life, and the quality of the variable geometries of the machined surfaces. This means that the introduced improvement involving the use of a ceramic grinding wheel with a radial outline makes it possible to carry out the grinding process of shaped surfaces with a radial curvature outline rather than only grinding surfaces with a shape resulting from the geometry of the conical outline of the ceramic grinding wheel.

## 5. Conclusions

The research work resulted in the following specific conclusions:

1. The conducted research has shown that the use of a dual-tool grinding head enables the effective implementation of the rough and finish grinding processes in a single fixture.
2. Analyses of the geometric structure of the machined surface showed a gradual reduction in the values of roughness parameters in two stages (after rough grinding and then finishing grinding) relative to the surface after the shaped milling process. In the case of the *Sa* parameter, the values were 5.30 μm, 1.12 μm, and 0.63 μm, respectively, for the process carried out with a conical grinding wheel and 2.10 μm, 1.73 μm, and 1.48 μm for the process carried out with a radial grinding wheel.
3. The value of the *Sds* parameter in the three stages was 393 pks/mm^2^, 563 pks/mm^2^, and 1203 pks/mm^2^, respectively, for the process carried out with a conical outline grinding wheel and 654 pks/mm^2^, 678 pks/mm^2^, and 686 pks/mm^2^ for the process carried out with a radial outline grinding wheel. Both cases showed an upward trend in changes.
4. The analysis of the Abbott-Firestone curve of the machined surface expressed by the *Spk* parameter showed a favorable reduction in the height of the tips in successive machining stages of 6.16 μm, 0.77 μm, and 0.33 μm, respectively, for the process carried out with a grinding wheel with a conical outline and 2.85 μm, 2.23 μm, and 2.09 μm for the process carried out with a grinding wheel with a radial outline.
5. Analysis of the impact of changing the shape of the ceramic grinding wheel showed that changing the axial outline of the ceramic grinding wheel makes it possible to significantly expand the range of applications of the dual-tool head without adversely affecting the quality of the machined surface.
6. Using a ceramic grinding wheel with a shaped radial outline allows greater control of the cross-sectional area of the grinding layer for different contours of the shaped surface.
7. The described research was a preliminary stage, which in the future will be used to determine the most favorable machining conditions with the developed dual-tool head. In future studies, the authors will focus on examining how the grain size of the ceramic grinding wheel and coated tool, changing the *a_e_* and *v_f_* parameters, as well as changing the trajectory of the machining paths, affect the grinding process.

## Figures and Tables

**Table 1:** Technical information on the abrasive tools applied.

**Ceramic Grinding Wheel**						
**Type**	**Size [mm]**	**Abrasive Type**	**Abrasive Grain Size**	**Grade**	**Structure**	**Bond Type**
3611	110 × 24 × 55	99A	60	K	9	V
		Electrocorundum	Medium	Soft	Open	Vitrified
**Flexible Grinding Wheel**						
**Type**	**Size [mm]**	**Grade**		**Material**	**Mounting**	
Trizact^®^	50	A45 P400		237 AA	Roloc	

**Table 2:** Workpiece material characteristics.

Material Number	Norm	Chemical Composition
1.2510	DIN EN 100 MnCrW4 AISI 01 PN NMWV	C: 0.90–1.05% Cr: 0.50–0.70% Mn: 1.00–1.20% P: max. 0.035% Si: 0.15–0.35% S: max. 0.035% W: 0.50–0.70% V: 0.05–0.15%

**Table 3:** Milling and grinding process parameters using a dual-tool grinding head with a conical outline grinding wheel.

Milling with a Ø20 mm Ball Mill		Grinding with a Conical Ceramic Grinding Wheel		Grinding with a Flexible Grinding Wheel with Trizact^®^ Grains	
*n*	3900 rpm	*n*	8100 rpm	*n*	15,000 rpm
*v_f_*	600 mm/min	*v_f_*	1000 mm/min	*v_f_*	1000 mm/min
v_c_ *	0.0–4.1 m/s	v_c_ *	30.0–46.6 m/s	v_c_ *	0.0–39.3 m/s
*a_e_*	1.0 mm	*a_e_*	2.0 mm	*a_e_*	2.0 mm
*a_p_*	1.0–5.0 mm	*a_p_*	0.04 mm	*a_p_*	0.1 mm

* Range of values in which the minimum value represents the minimum radius of the tool and the maximum value represents the maximum radius of the tool.

**Table 4:** Milling and grinding process parameters using a dual-tool grinding head with a radial outline grinding wheel.

Milling with a Ø20 mm Ball Mill		Grinding with a Radial Ceramic Grinding Wheel		Grinding with a Flexible Grinding Wheel with Trizact^®^ Grains	
*n*	3900 rpm	*n*	8100 rpm	*n*	15,000 rpm
*v_f_*	600 mm/min	*v_f_*	1000 mm/min	*v_f_*	1000 mm/min
v_c_ *	0.0–4.1 m/s	v_c_ *	41.5–46.6 m/s	v_c_ *	0.0–39.3 m/s
*a_e_*	1.0 mm	*a_e_*	0.5 mm	*a_e_*	2.0 mm
*a_p_*	1.0–5.0 mm	*a_p_*	0.04 mm	*a_p_*	0.5 mm

* Range of values in which the minimum value represents the minimum radius of the tool and the maximum value represents the maximum radius of the tool.

**Table 5:** Values of selected surface roughness parameters after milling (Stage 1), after milling and rough grinding (Stage 2), and after milling, rough grinding, and finish grinding (Stage 3) using a conical outline grinding wheel and a radial outline grinding wheel: *Sa*—arithmetic mean deviation of the surface; *St*—total height of the surface; *Sds*—density of summits of the surface; *Sq*—root-mean-square deviation of the surface; *Str*—texture aspect ratio of the surface; *Spk*—reduced peak height.

Parameter	Grinding Wheel	Stage 1	Stage 2	Stage 3
*Sa*	Conical	5.30 μm	1.12 μm	0.63 μm
	Radial	2.10 μm	1.73 μm	1.48 μm
*St*	Conical	31.80 μm	14.40 μm	6.72 μm
	Radial	65.00 μm	40.40 μm	18.30 μm
*Sds*	Conical	393 pks/mm^2^	563 pks/mm^2^	1203 pks/mm^2^
	Radial	654 pks/mm^2^	678 pks/mm^2^	686 pks/mm^2^
*Sq*	Conical	6.39 μm	1.46 μm	0.81 μm
	Radial	2.78 μm	2.29 μm	1.97 μm
*Str*	Conical	0.030	0.019	0.037
	Radial	0.008	0.003	0.042
*Spk*	Conical	6.16 μm	0.77 μm	0.33 μm
	Radial	2.85 μm	2.23 μm	2.09 μm

## Data Availability

The original contributions presented in the study are included in the article; further inquiries can be directed to the corresponding author.
